# Nineteenth Century Amorphous Calcium Carbonate

**DOI:** 10.1021/acs.cgd.4c01066

**Published:** 2024-11-11

**Authors:** Bart Kahr, Sophia Sburlati, Jackson Comes, John Mergo, Willem L. Noorduin, Jong Seto

**Affiliations:** † Department of Chemistry and Molecular Design Institute, New York University, 29 Washington Place, Silver Center, New York, New York 10003-6688, United States; ‡ Center for Biological Physics and School of Engineering of Matter, Transport, and Energy, Arizona State, Tempe, Arizona 85287-0002, United States; § AMOLF, Science Park 104, 1098 XG Amsterdam, The Netherlands; ∥ Van’t Hoff Institute for Molecular Sciences, University of Amsterdam, Amsterdam 1090GD, The Netherlands

## Abstract

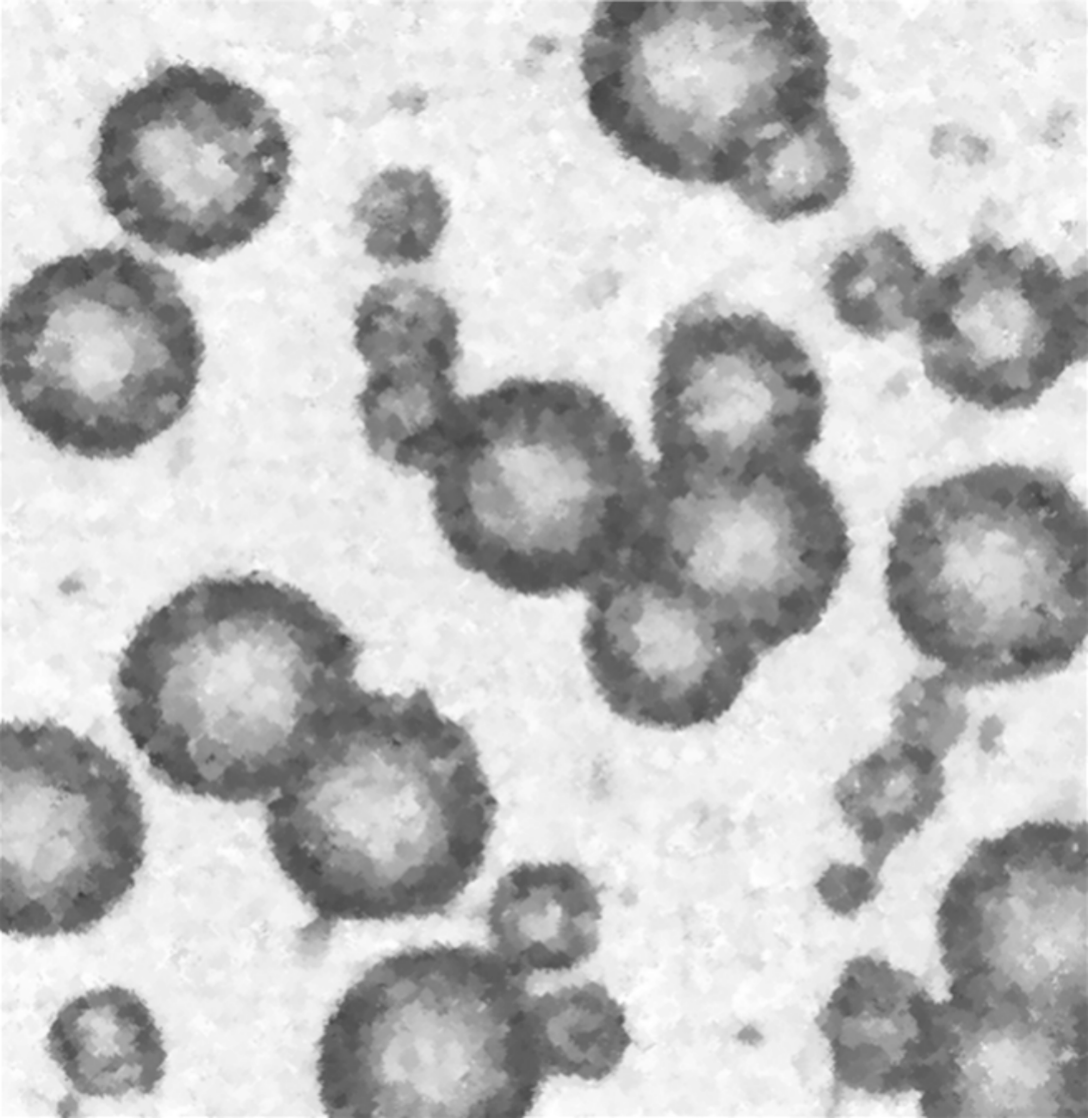

The work of the English anatomist George Rainey is compared with that of the Dutch naturalist Pieter Harting. While the latter is regarded as a pioneer in biomimetic inorganic crystallography for precipitating unusual crystallographic forms that mimic the products of living organisms, the work of Rainey largely has been forgotten. In fact, Rainey first prepared amorphous calcium carbonate, a material that can be molded by organisms to form biogenic crystals. Rainey’s extensive experimentation with amorphous calcareous bodies observed in a variety of organisms was at one time considered a significant and pioneering chapter in inorganic chemical morphogenesis and it should reclaim some of its former assessments. Rainey’s interpretations of crystal form and the effects of gravity on crystal growth mechanisms, however, are historical curiosities that should be left behind, except to the extent that they show how the efforts of an individual may appear diminished by the dynamic process of consensus building in science. Harting also prepared amorphous calcium carbonate, but more than a decade after Rainey. While Rainey was a quiet scholar with steady habits, Harting was a statesman, a champion of the down-trodden (albeit with prejudice), a popular educator, a temperance advocate, and a sci-fi novelist, in addition to being a professor. Harting’s public life may account for his outsized place in our collective memory. Rainey’s synthesis of amorphous calcium carbonate in the presence of gum arabic was repeated in a modern setting. Microspheres were characterized by scanning electron microscopy, established as hollow by X-ray microtomography, and were shown to have the composition of calcium carbonate by energy dispersive X-ray analysis. They were amorphous by powder X-ray diffraction.

## Abstract 2

George Rainey (1801−1884), an anatomist at St. Thomas’s Hospital in London, first described the preparation and crystallization of amorphous calcium carbonate.

## Overview of Ward’s Biopathological Crystals

More than three decades ago, Michael D. Ward led researchers in applying atomic force microscopes (AFMs) to the kinetics of the *in situ* growth of crystals that form biominerals. Previously, researchers sketched crystal growth dynamics from static electron micrographs. These were superseded by moving pictures of AFM step propagation.^1−3^ Ubiquitous calcium carbonate polymorphs, among the most common structural biominerals, were a focus of scanning probe microscopists.^4−6^ Ward, on the other hand, focused on biopathological crystals,^7^ foremost kidney stones, a worthy target in the estimation of anyone who has had the misfortune to grow one *in vivo*. Ward’s first papers in this area tackled the most common kidney affliction, calcium oxalate stones,^8−11^ and the macromolecules that affect their growth by adhesion. In 2010, he launched a series of investigations into rarer and more pernicious cystine stones.^12−15^ A small number of persons are stricken with frequent and painful kidney stones due to their inability to metabolize the amino acid cysteine (reduced cystine).^16^ Ward identified the rate determining substeps in the spiral growth of hexagonal cystine crystals^17^ and designed cystine-imposter molecules that function as drugs to diminish kidney stones by inhibiting crystal growth.^18−20^ Later, he analyzed pharmaceutical crystalline precipitates that he named *xenostones*.^21^ He again resorted to kinetic and morphological analyses by *in situ* AFM. Ward’s trajectory followed progressively rarer kidney stones. He never studied carbonate stones, however. Calcium carbonate stones are rare in people, somewhere between 0.01% and 1% depending on the source of the data.^22^

About the same time that Ward refined the use of *in situ* AFM, three decades ago, our view of calcium carbonate took on a new dimension. Amorphous calcium carbonate (ACC) increasingly was recognized as essential in the ability of organisms to grow single crystals of calcium carbonate with atypical morphologies that were not accounted for by classical crystal growth mechanisms. While occasionally ACC had been the subject of sporadic study,^23−25^ surely it was overlooked often because it is indifferent to the polarization state of light, and free of Bragg scattering of X-rays. Pioneering work on calcitic sea urchin spine development in 1997^26^ set the stage for the forecast in 2003 that ACC, as “a transient precursor phase for calcite or aragonite, may have far-reaching implications in the field of biomineralization” and may “radically change the manner in which we understand biological calcium carbonate.”^27^ Only after the these bold predictions–that have indeed come to pass^28−32^ – did researchers use the AFM to study the crystallization of ACC.^33−37^ Comparatively recent publications give overviews of the water-dependent transitions from ACC to crystalline calcite from the perspective of the most incisive scattering experiments,^38,39^ and by simulation.^40^

## Predecessors

Macromolecules play important roles in affecting the growth in biologically relevant crystals.^41,42^ Some of the early speculations on crystal interactions with biological extracts come from Pieter Harting (1812–1885)^43^ whose work we first learned about in Geoffrey Ozin’s influential 1997 *Accounts of Chemical Research* article.^44^ (Incidentally, Ozin learned of Harting from our late NYU colleague Nadrian Seeman (1945–2021).^45^ More incidentally, Harting’s father died of bladder stones when Pieter was just seven years old. This loss was surely formative, but we cannot say if it was scientifically formative.)^46^ Ozin credited Harting as the pioneer in an up-and-coming discipline: “During the course of our research on inorganic morphosynthesis,” wrote Ozin, “we became aware of the creative experimentation of Professor Pieter Harting, who, at the end of the 19^th^ century, took the first steps in the field that we now call biomimetic inorganic chemistry.”^44^ This account was a source for Philip Ball in his vivid history of pattern formation in nature.^47^ Harting described recipes for coaxing calcium carbonate into complex morphologies involving the diffusion of bile, blood, mucus and even chopped-up oysters^43^ with soluble salt precursors. Today, fabricating oddly shaped calcium carbonate crystals from solutions containing macromolecules has become an active occupation of crystal growers,^48−50^ motivated by the great advances of the past generation in our understanding of how organisms grow crystals, biomineralization.^51,52^ It is in this context that Harting is of greatest interest to contemporary scientists and engineers and many have become newly acquainted with him through the writings of Ozin and Ball.^53^

Ozin and Ball relied on D’Arcy Thompson’s (1860–1948) *On Growth and Form*,^54,55^ in which Harting’s work was analyzed in detail. However, Thomspon invariably mentioned Harting with another scientist, George Rainey (1801–1884, Figure 1). “Let us return,” wrote Thompson, “. . .to the general subject of the forms assumed by certain chemical bodies when deposited or precipitated within the organism, and to the question of how far these forms may be artificially imitated or theoretically explained. Mr George Rainey, of St Thomas’s Hospital...and Professor P. Harting, of Utrecht, were the first to deal with this specific problem...” Thompson continued, “Rainey and Harting used similar methods—and these were such as other workers have continued to employ...” While these remarks occurred almost two-thirds of the way into Thompson’s 1100-page book, Rainey first appeared on page 10 for having made a prescient remark that is perfectly suited to Thompson’s purpose. “[I]t is illogical,” Rainey is quoted in a note about artificial calculi, “to suppose that in the case of vital organisms a distinct force exists to produce results perfectly within the reach of physical agencies...”^56^ Rainey’s work appeared in two additional articles^57,58^ and a book, *On the Mode of Formation of the Shells of Animals, of Bone, and of Several Structures by a Process of Molecular Coalescence* (1858).^59^

**Figure 1:**
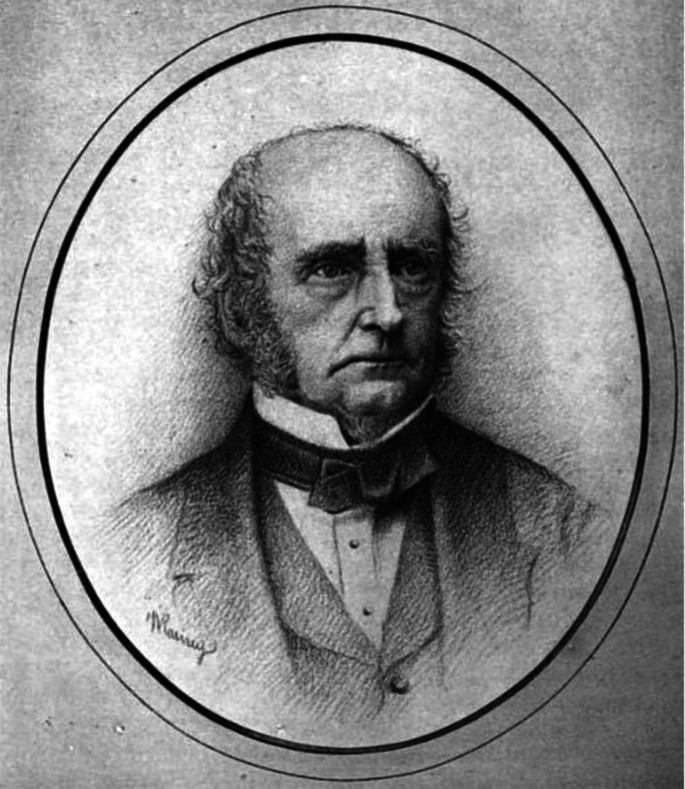
Charcoal portrait of George Rainey by his son, William, as reproduced in ref (74). This material has been provided by The Royal College of Surgeons of England. The original may be consulted at The Royal College of Surgeons of England.

A more enduring work of anatomy was published in London, also in 1858, that of Henry Gray (1827–1961).^60^ It might have earned the fuller nickname, “Gray’s and Carter’s Anatomy” in honor of its illustrator, Henry Van Dyke Carter (1831–1897),^61^ but apparently Gray diminished the contributions of Carter, and even interfered with Carter’s receipt of royalties due.^62^ Carter was also a medical doctor, but learned the craft of illustration from his father, a professional artist. Carter, after completing his work on “Gray’s Anatomy,” and frustrated by Gray’s shabby treatment of him, went to Bombay as a member of the Indian Medical Service and published, in addition to works on leprosy and elephantiasis, a treatise on the *The Microscopic Structure and Mode of Formation of Urinary Calculi*,^63^ a forerunner to the opus of Ward honored here. Throughout, Carter, like Thompson, acknowledged the contributions of Harting *and* Rainey to his interpretations of polycrystalline patterns and nonpolyhedral forms. He gives pride of place, however, to Rainey who “is due, so far as the author knows, the credit of fully demonstrating the existence and character of that widely operating process termed by him ‘molecular coalescence,’ which the author here only attempts to apply in explanation of the construction of certain calculous ingredients in man.”^63^

Ambivalence toward Harting is further articulated by one of Rainey’s St. Thomas students, William Miller Ord (1834–1902). He chastised Harting for ignoring Rainey’s work on “Synthetical Morphology” which has “not met with the notice and appreciation it deserves.”^64^ Ord, in his treatise *On the Influence of Colloids on Crystal Growth and Cohesion*,^65^ later softened. In reference to Harting’s summary of a full accounting of crystallization,^43^ Ord said, “As regards this work I am bound to recant some observations which I made. . .to the effect that to those who were acquainted with Mr. Rainey’s work it offers little that is new. I am bound, on reperusal, to acknowledge that it is deeply interesting, full of important facts, and most suggestive.”^65^ Still, to those in England, Harting was a latecomer, virtues notwithstanding.

Unforgiving was George Busk (1807–1886), one-time editor of the *Quarterly Journal*.^66^ Referring to Harting’s article,^43^ Busk laments that

[I]t is strange to see...in the pages of the ‘Microscopical Journal,’ a paper on the subject in question, in which the name of Mr. George Rainey does not appear, to whom alone, so far as I know, is all originality with regard to it due. Many years since (1857–1861) Mr. Rainey’s observations on the formation of globular crystalline masses of carbonate of lime, etc., in mucilage of gum arabic, and other fluids containing organic colloid matter, were. . . the results of numerous carefully conducted experiments, and are filled with highly ingenious and suggestive observations and remarks, well worthy of more attentive consideration than they have as yet received. So far as I can see, there is nothing in Professor Harting’s “preliminary communication,” including the figures, which may not be found in Mr. Rainey’s papers. If Professor Harting, as seems scarcely possible, should be unacquainted with the labors of his predecessor, it is as well he should become so before the publication of his memoir in extenso, when, I have no doubt, he will do full justice to Mr. Rainey.^66^

### Rainey Was Overlooked, *In Extenso*

The indignation persisted for decades. The Toronto-based surgeon, James Crawford Watt in 1923, wrote: “Harting has been erroneously credited by many writers with the discovery, in 1872, of the calcospherites [see below] formed in colloidal solutions. Rainey described and gave accurate illustrations of these bodies in 1857, and had observed them as early as 1849, and so deserves the credit of this discovery.”^67^

Rainey seemed to have gone from fiercely defended to mostly forgotten. Ozin and co-workers originally introduced Harting to contemporary readers in a “Talking Point” editorial in 1995,^45^ emphasizing the wages of overlooking our predecessors. Here, Ozin and co-workers acknowledge that Rainey and Harting were paired by Thompson, but Harting is solely designated as the “'overlooked father' of our field”. Stephen Mann offered a spirited rebuttal to Harting’s purported anticipation of *bona fide* biomineralization,^68^ arguing that there are vast differences between simple *in vitro* mechanisms and *in vivo* control. Of course, the relevance of laboratory experiments as proxies for complex processes in organisms is almost always *in part*. Accessible models are instructive if not perfect simulations. Mann followed Thompson and mentioned Harting *and* Rainey, as did Ozin in his reply, but they both left Rainey by the wayside in their subsequent discussions. Ozin cited Harting’s originality as “the common thread that runs through all modern syntheses of inorganic materials inspired by biological construction principles.”^69^

Rainey appreciated that artificial mechanisms indeed can inform natural ones, if imperfectly. They give us bright foothold. There is no credible reason to take exception to this way of working in science. Rainey wrote, “[A]rtificial products cannot fail to throw great light upon the genesis of the natural ones, and thus tend to emancipate this department of histology from the obscurity in which it now lies, and bring it under the domain of experimental physical science.”^59^

Still, selective–if unintendedly selective–hagiography is persistent. A most recent history of biomineralization acknowledges Harting but not Rainey,^70^ while elsewhere Harting solely is recognized as a “founding father” of biomineralization in a Harting-like study of ACC grown in the presence of ovalbumin.^71^ “What started with Harting...” writes Antonietti and Fratzl, “has turned into a broadly applicable set of thoughts and principles which inspires practically the complete breadth of material science.”^72^ Our friend described Harting as the “forgotten Dutch zoologist,” but he could have been more forgotten as was Rainey.^73^

Thompson remembered Rainey as “a man of learning and originality.”^54^ As a demonstrator of anatomy at St. Thomas’s, “he followed that modest calling to a great age, and is remembered by a few old pupils with peculiar affection.”^54^ Another former student, William Warwick Wagstaffe (1843–1910) remembered Rainey’s powerful moral influence that extended to generations of students.^74^ By what circumstance did a learned, original, modest and moral investigator, remembered with great affection become detached from Harting, the pioneer of biomimetic inorganic chemistry? Why was Rainey’s work, at best, “spasmodically applauded down the years?”^75^ We aimed to give Ward answers.

## George Rainey

### Biography^74,76^

George Rainey (1801–1884) was born in Spilsby, about 200 km north of London. He was apprenticed to a doctor and aspired to become one. Rainey, of modest circumstances, was ridiculed for his ambition. He resolved to win over his critics by studying assiduously. In 1824 he entered St. Thomas’s Hospital as a student. To meet expenses, Rainey worked as a “grinder” preparing other students for their examinations. Wagstaffe described medical students of the time as Bob Sawyer types, referring to Charles Dickens’s character in the *Pickwick Papers*.^77^ Rainey earned a Royal Society of Chemistry qualification while continuing his work as a grinder. Exhausted, and perhaps suffering from consumption, Rainey took leave for the continent, returning in good health after 5 years. He was appointed Demonstrator of Human Anatomy and the Microscope in 1846, a position he held until his death at age 84. As an octogenarian, his duties became optional, receiving a government pension for his services to science.

### *Spherules* of Carbonate of Lime

In 1849, Rainey, by his account,^59^ was motivated to distinguish with the optical microscope chemical bodies produced by living organisms–for example “globular calculi found in the urine of some quadrupeds” – and similar compositions prepared *in vitro*. For verisimilitude, he sought to mimic organisms in the laboratory with some “viscid animal or vegetable substance, such as albumen or gum arabic.” Harting operated along similar lines and emphasized albumen (see later) whereas Rainey favored gum arabic.

Gum arabic is the hardened sap of acacia trees. It is composed of glycoproteins as well as polysaccharides of arabinose and galactose.^78^ The morphological influence of natural resins on crystal growth can be fantastic and we have been trying to identify the component compounds of resins in some cases that have the greatest influence as additives. Abietic acid is the most abundant resin acid in Canada balsam and affects crystal growth as well as does the complex mixture.^79−82^ Gum arabic also has been shown to stabilize vaterite a metastable form of crystalline calcium carbonate.^83^

Rainey observed globular forms (aka *spherules*) of transparent calcium carbonate precipitated between microscope slides after contacting two solutions of gum arabic, one containing potassium bicarbonate and the other calcium malate. He communicated this result to several friends.^57^

Thirty years earlier, also at St. Thomas’s hospital, Alexander Marcet, a physician and chemistry lecturer, announced the analysis of a human gall-bladder stone made almost entirely of calcium carbonate (previously found in animals).^84^ Rainey may have been aware of the famous mass discovered at his St. Thomas Hospital. The importance of these observations, however, was not impressed fully upon Rainey until 1856 when he, too, observed similar products in organic tissue. This observation gave Rainey a rousing purpose: “Now, as the perfect resemblance of the globular form of the carbonate of lime, as prepared artificially, and as occurring in nature, indicates a corresponding similarity in the nature of the process by which they are formed, and an identity of the forces concerned in their formation,” a study of the modes of formation is requisite.^59^

Rainey was struck by his spheres of newly precipitated carbonates as well as by their tendency to aggregate. He emphasized the evolution of his mixtures^58^ and wrote in his monograph:

I have been anxious to extend and improve my former process for obtaining the globular form of carbonate of lime by making the conditions more like the natural ones, and by so performing the experiments that the changes, which the carbonate undergoes in its passage from an apparently amorphous state to large globules, may, as they are taking place, allow of being examined by the microscope.^59^

To account for the observation of the aggregation of spherules, he turned from his gum arabic to the heavenly bodies. He identified gravity as the agent responsible for ACC particles and their behavior. This judgment is far from the mark, even given its time. Therefore, we quote an extended passage that exposes Rainey’s logic.^59^

As every particle of matter, whatever may be its form or dimensions, is admitted by philosophers to be under the influence of gravity, to which law, if universal, the molecules of carbonate of lime, as produced in the manner already described, can form no exception, it must follow that, the instant they are brought into existence, they will commence arranging themselves in spherical figures, unless there should be some other force of an opposite kind acting upon them, adequate either entirely to overcome that of gravity, or sufficient only imperfectly to resist its influence; in which case results of an intermediate kind would be produced, depending upon the relative powers and modes of operation of the opposing agencies. Now, as it is an undoubted fact, and one admitting of ocular demonstration, that the particles of carbonate of lime formed by the double decomposition of a salt of lime and carbonate of potash previously dissolved in a solution of vegetable gum, or of albumen of about the same density as the resulting carbonate, do assume, as their first appreciable form, that of minute spherules, and as this is exactly the figure which the molecules composing these particles would assume under the mechanical conditions in which they are placed, if they were simultaneously subjected to the effective influence of gravitation, that is, if they attracted one another with a force varying inversely as the squares of the distances between them, and directly as the quantity of matter in each molecule, the sphericity of these particles may be inferred to be the effect of gravity. And, besides, as there is no other known agency which could produce the same results under the same circumstance, this fact ought not only to be looked upon as the effect of gravity, but also as a proof of it.

We are reminded of Dawkins’s rejection of Bishop Montefiore who failed to see any reason that polar bears should develop a white coat through evolution and natural selection. Dawkins instructs that the argument based on “personal incredulity” is of little merit in science.^85^ Rainey asserts that there is “no other known agency which could produce the same results.”^59^ No other agency *known to him*. Rainey gives few quantitative statements. The gravitational attraction between one-micron calcium carbonate spheres separated from one another by one micron is about 10^–28^ N, a very small force indeed.

Rainey contemplates the role of electric and magnetic forces but in his view they would be subservient to the action of gravity. Maxwell was just beginning his ascent while Newton had reached the zenith of his English orbit. We now appreciate that surface energy minimization leads to spherical particles naturally, as is the tendency of ACC to form dense liquid precursors,^86^ and depletion forces can drive aggregation entropically.^87^ In fairness to Rainey, in 1858, colloid science, was in its infancy. Just the previous year, Faraday reported a colloidal solution of gold.^88^ Graham is credited with elevating the science of colloids to a separate status, but his definitions were published only in 1861, the year he coined “colloid” from the Greek for glue-like.^89^

Rainey’s persuasion was not helped by his prolixity. He said the same thing over and over in massive paragraphs assembled from long sentences. This style is in evidence in some of the passages quoted here. Rainey’s writing can be attributed only in part to the manner of composition in the mid-19th century. He strained the excesses of his time.

Contemporary biomineralogists^27,30^ attribute the first mention of ACC to Edward A. Minchin (1866–1915) who wrote about young calcareous spicules that do not “light up”^90^ between crossed nicols, evidence that they were optically isotropic. It seems clear that in the waning years of the 19th century, scientists were recognizing the role of ACC in biogenic spicule growth, a fair claim to the discovery of ACC. In 1993, researchers were sensitized to anomalously weak diffraction from sea urchin spines that were presumed to be single crystals,^91^ putting Minchin’s observations on a solid analytical foundation. The work of Rainey, however, must reset the first observation of ACC by two generations.

**Figure 2:**
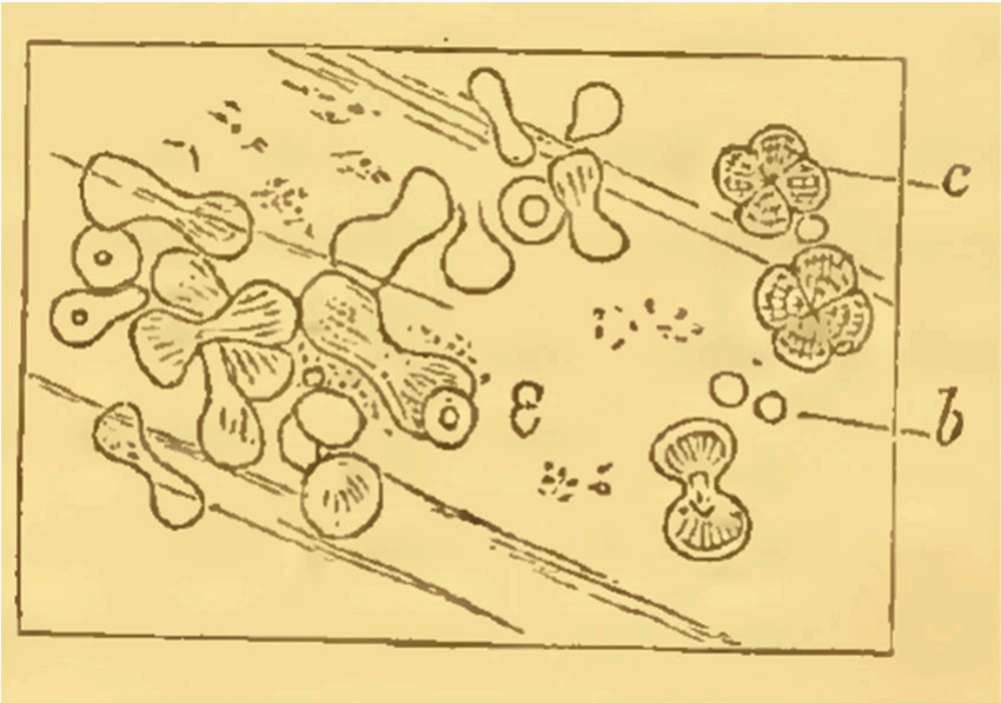
Detail of Rainey’s Figure 6 of an oyster shell (ref (59)). *b* marks globules of amorphous carbonate of lime. *c* marks distinct bodies “in which a cross can be seen with polarized light.” This material has been provided by King’s College London. The original may be consulted at King’s College London.

Rainey’s monograph is divided between its Physical Part, and the more extensive Physiological Part. Rainey was convinced that the bodies he observed *in vitro* with gum arabic were amorphous spherules of carbonate of lime not fundamentally different from those found in nature. For instance, in a detail of his microscopic analysis of an oyster shell, Figure 2, Rainey identifies amorphous globules marked as *b*.

Rainey’s ACC was a transitory species that would ultimately lead to crystals, often radial or spherulitic:

This singular *transformation* of a condition of carbonate of lime, which, when in small pieces, was perfectly globular and appeared to be completely homogeneous, into an imperfectly crystalline structure after these same particles had become incorporated into large spherical masses, will require now to be considered, and the cause of the change from the globular into the crystalline form explained. [emphasis added]

Rainey used the word “amorphous” frequently. It is important that he meant what we mean today, lacking long-range crystalline order. We can only take him at his word, “if there ever was a period when matter existed unacted upon by attraction or impulsion, it must have been in a chaotic or amorphous state–a something “without form.”^92^ Rainey also frequently uses “globular” to describe his precipitates. The latter is a word that contemporaries might associate with lack of structure (“A glob of...” e.g.) but Rainey used it as meaning spherical, and more particularly as globe-like, since gravity was his animating force. Sometimes his globules were amorphous, sometimes crystalline. Despite his expert use of the polarized light microscope, because he was looking at calcium carbonate solutions adulterated by gum arabic, he conceded “how far these [globules] ought to be looked upon as crystals, I shall not attempt to decide.”^58^ Generally speaking, his process of coalescence proceeded from nebulous and amorphous, to amorphous and globular, to globular and spherulitic.

A comparable process of phase separation and aggregation of calcium carbonate in the presence of organic matter was given a name and an acronym generations later, the polymer-induced liquid-precursor (PILP) process.^93^ More recently, researchers have recast the PILP acronym as CAT (colloid assembly and transformation).^94^ The PILP chemists, emphasized that transitory ACC was not observed *in vitro* until almost the 21st century but Rainey asserted that, “Nothing can be more dissimilar in appearance than the different forms assumed by the globular carbonate of lime in the various parts of the same shell, and it is only by examining the passage of the one form into the other that their identity can be determined.”^59^ Rainey’s diagram of “molecular coalescence” in Figure 1 of ref (57), a process of calcium carbonate precipitation, aggregation, and crystallization, compares favorably with later drawings (see Figure 2 in Gower and Odom^93^). Researchers recently showed that polysaccharides not unlike those in Rainey’s gum arabic can produce 10 nm ACC particles that grow by accretion.^95^

The long-term stabilization of ACC was prioritized by 21st century materials science researchers.^96−99^ Arguably the most dramatic stabilization and transformation of ACC remains the demonstration of Aizenberg et al.^100^ Rainey had followed the nuclei of amorphous globules of calcium carbonate that remained unchanged for three to four months.^58^

For completeness, we state that amorphous dolomite CaMg(CO_3_)_2_ was described by 1866 by Thomas Hunt (1826–1892).^101^ The role of Mg^2+^ ions in stabilizing ACC has been long a subject of study.^102^ That said, dolomite is not calcium carbonate; dolomite’s role in biomineralization is still a mystery under active investigation,^103^ and it is best held at arm’s length for brevity here.

### Remaking Rainey’s Amorphous Calcium Carbonate

In 1962, A. J. Harding Rains (not to be confused with Harting or Rainey) reviewed the histology of gall stones over a period of >200 years.^75^ He compared successive generations of investigators, covering similar territory but each reaching further with the passage of time.

Rains admired Rainey and reproduced the latter’s synthesis of spherules of calcium carbonate. Rains instructions, more straightforward than Rainey’s, are copied.

Rainey’s simple experiment was to use two filtered solutions of gum arabic of different specific gravity, one of which was saturated with carbonate of potash [and the other with calcium malate]. The heavier solution was put into a glass phial, and the lighter solution carefully poured on top, so that the solutions were mixed as little as possible. The phial was kept for three to 6 weeks, and a glass slide which had been laid in the direction of the long axis of the phial was examined under the microscope.^75^

**Figure 3:**
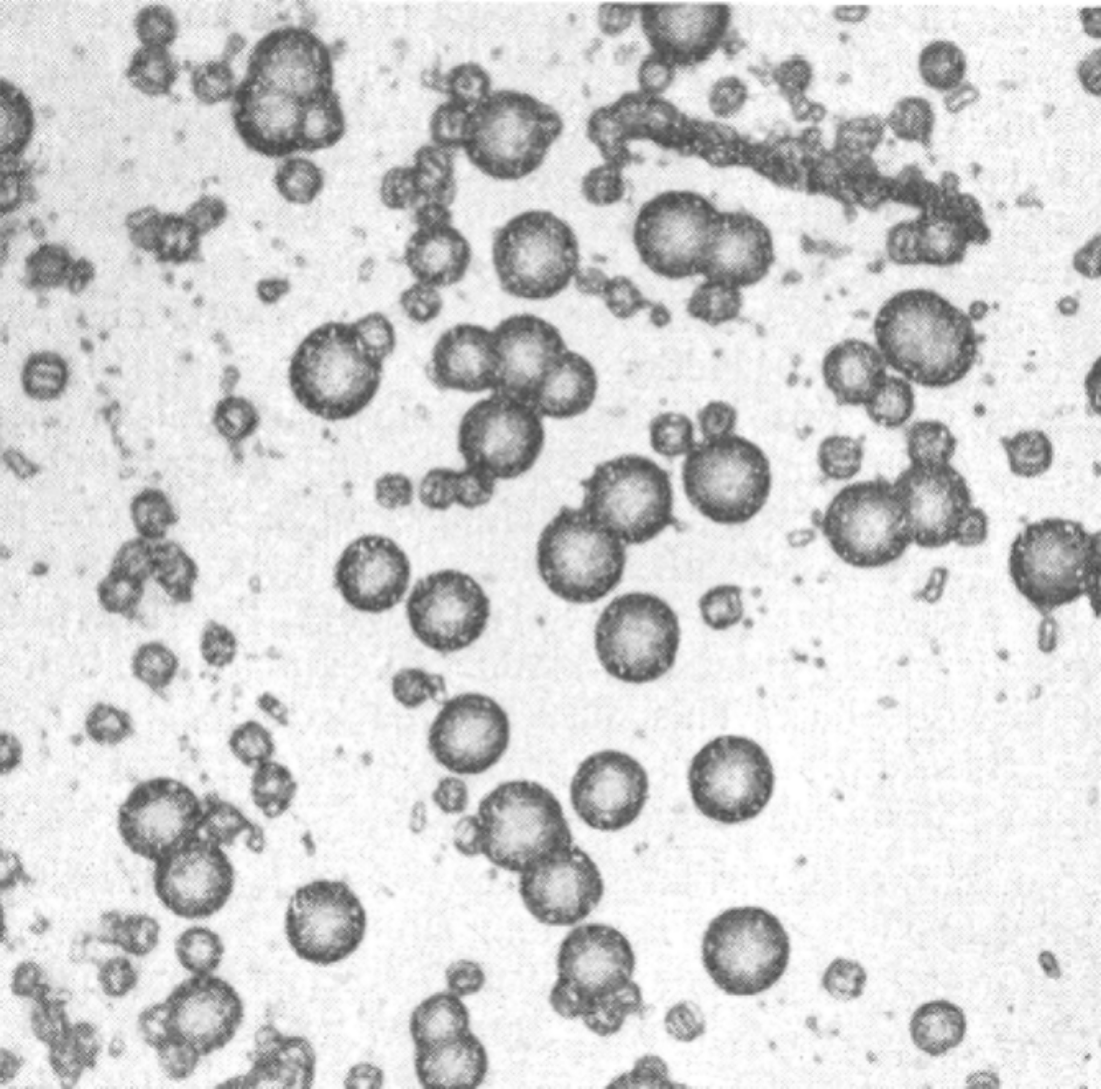
“Spherolith formation of calcium carbonate by layering of gum acacia showing the growth of spheroliths by aggregation. (×110.) (Rainey’s experiment reproduced, 1962).” From Figure 6 of ref (75). Reprinted with permission 5896480774933. Copyright 1962 British Medical Journal Group.

The particles produced in this way are shown in Figure 3. They do indeed resemble ACC, subsequently observed by many. But Rains did not characterize his spheres by any chemical or physical method. Here, we repeat this work once more, supported by modern methods of analysis, to confirm that Rainey did indeed form ACC phases in the middle of the 19th century as did Rains in the middle of the 20th century.

**Figure 4:**
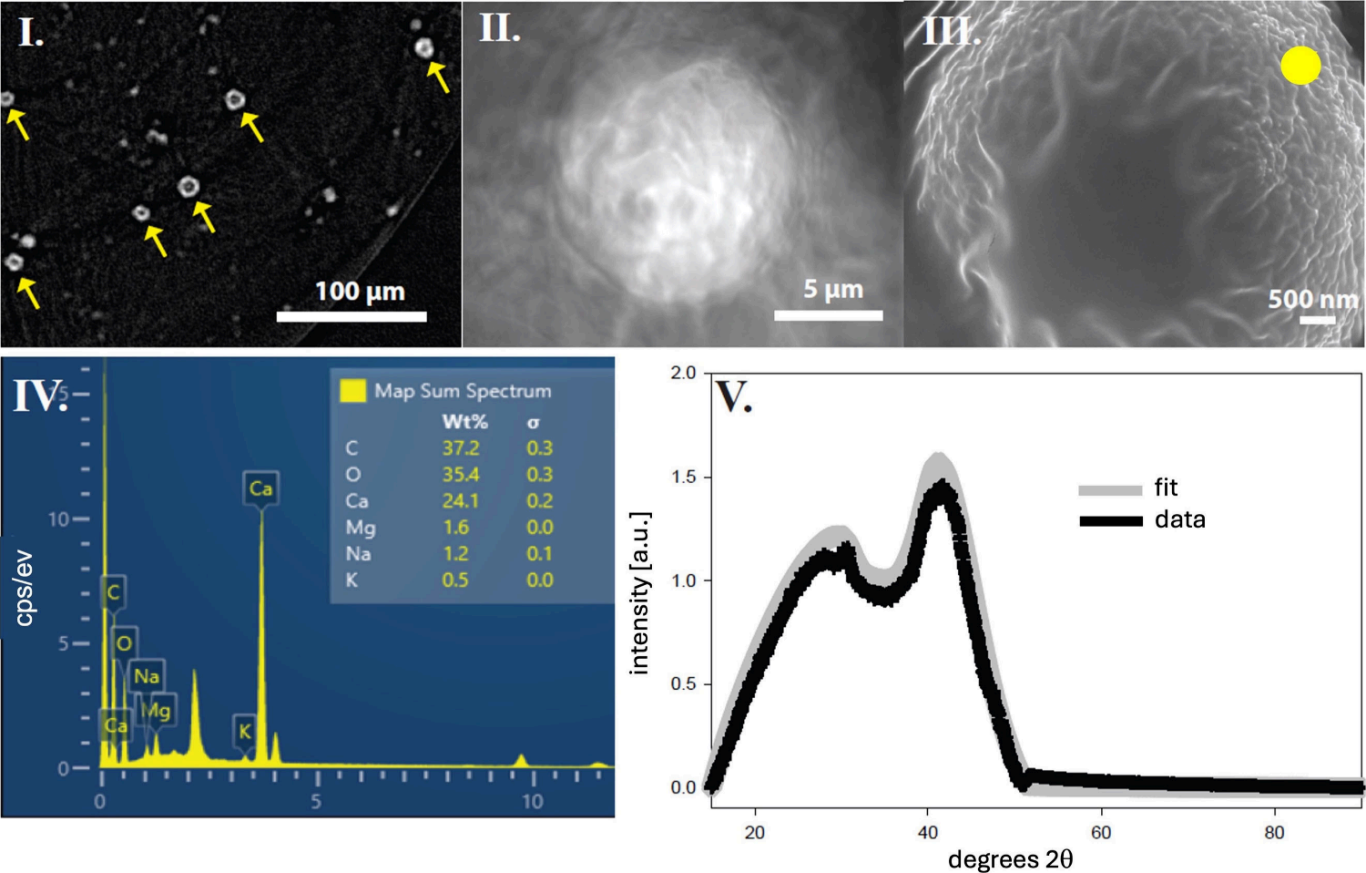
Rainey’s amorphous calcium carbonate (ACC) spheres. I. Synchrotron X-ray computer tomography (CT) of the reaction tube containing Ca-malate and K_2_CO_3_ gum arabic [yellow arrows indicate forming ACC spheres]. II. Nanosynchrotron-X-ray CT of a single ACC sphere. III. Scanning electron micrograph of a single ACC sphere. IV. Energy dispersive X-ray (EDX) spectroscopy of ACC sphere [see yellow spot for location in III]. V. X-ray diffraction of the ACC spheres.

Like the protocols of Rainey (and subsequently Harting) we utilized pure gum arabic medium (W471, Holbein Gum Arabic, Holbein Works, Ltd. Osaka, Japan) to compose a 100 mM Ca(C_2_H_4_O(COO)_2_) (Santa Cruz Biotechnology, Inc., Dallas, TX USA) stock solution, and a 100 mM K_2_CO_3_ (Sigma-Aldrich, St. Louis, MO USA) stock solution, whereby equivolumes of the two are aliquoted into a 15 mL Falcon tube without mixing. After an incubation period of 48 h at room temperature and ambient pressure, a white precipitate was observed at the interface of the two solutions. This precipitate was gently aliquoted from the reaction tube and analyzed with X-ray diffraction (Miniflex 600, Rigaku Corp, Tokyo, 196-8666 Japan) as well as high resolution imaging methods (Figure 4). The viscosity of the gum arabic mediates the diffusional mixing of the Ca(C_2_H_4_O(COO)_2_) and K_2_CO_3_ such that the predominant species is CaCO_3_. We confirm that these ACC spherules are also hollow initially most perhaps due to the earlier formation of smaller ACC constituents and stabilization at local interfaces of the gum arabic in a similar fashion to the formation of Pickering emulsions.^104,105^

We see a distribution of these hollow ACC spheres in X-ray tomographs (BL 8.3.2, ALS, Lawrence Berkeley Laboratory, Berkeley, CA 94720 USA) in the size range of 5–10 μm in the gum arabic media (Figure 4I). At higher spatial resolution with X-ray nanotomography (BL 6.2, SPEAR, Stanford Linear Accelerator Center, Stanford, CA 94025 USA), we can see these spheres are wrinkled at the surface (Figure 4II) by the imperfect accumulation of smaller spherules by drying during sample preparation. Rainey described the spheres on first agglomeration as mulberry-like or like the glomeruli of pathologists.^59^ This was further confirmed with scanning electron microscopy (SEM) and energy dispersive X-ray spectroscopy (EDX) (Auriga, Zeiss AG, Oberkochen, 73447 Germany). The elemental composition of CaCO_3_ in Figure 4IV was established at the yellow spot in the SEM in Figure 4III. The amorphous state of CaCO_3_ is confirmed by the broad, X-ray diffraction patterns with the absence of calcite Bragg peaks (Figure 4V).

## Pieter Harting^46^

### Biography

**Figure 5:**
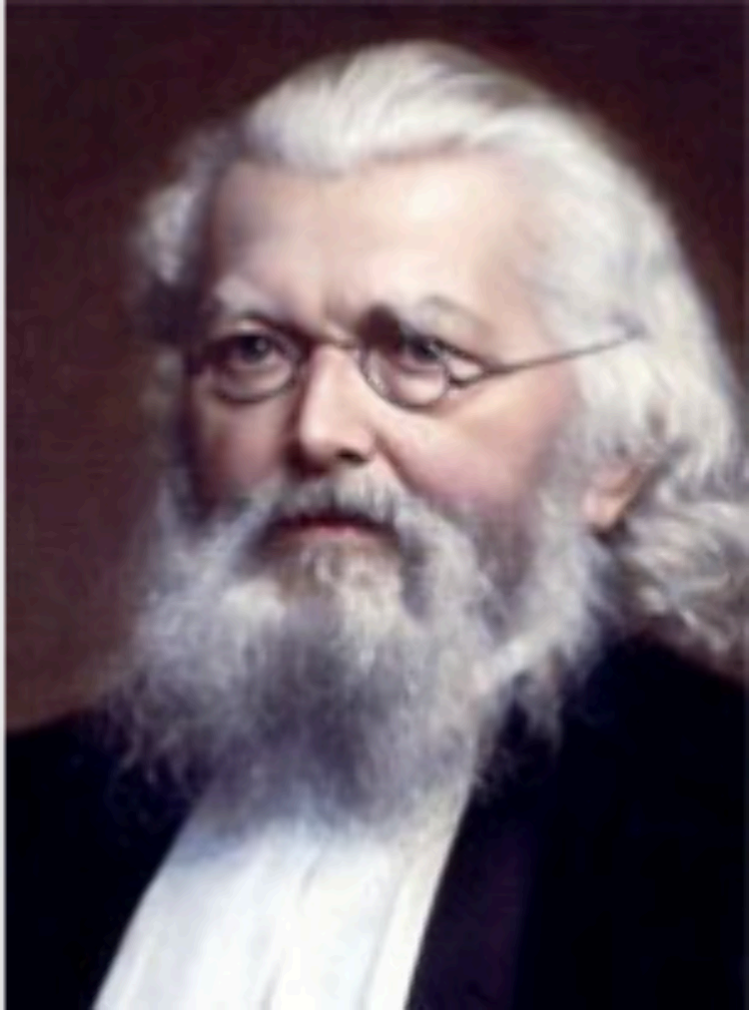
Oil painting of Pieter Harting by Johan Heinrich Neuman (1819–1898) (*Professoren van de Universiteit Utrecht*). Universitätsmuseum Utrecht. This work is in the public domain in its country of origin and other countries and areas where the copyright term is the author’s life plus 100 years or fewer.

Pieter Harting (Figure 5), born in Rotterdam, was 11 years younger than George Rainey, but the researchers had parallel professional lives. Like Rainey, Harting earned a medical degree (in 1835 from the University of Utrecht). He practiced medicine for a brief time and taught medicine in Franeker, before returning to Utrecht where he served as professor of pharmacology, plant physiology, and zoology. Like Rainey, Harting was an early champion of the theory of Darwin’s theory of evolution. Like Rainey, he had an enduring fascination with the optical microscope and taught microscopy^106^ to generations of students. Harting lost one son in infancy, and his two adult daughters during the same year while retiring from his long academic career in Utrecht.

We know much more about Harting’s life from his fulsome autobiography^46^ than we do the life of Rainey. For instance Harting gives an anecdote about an inspiring carbonate crystallization experiment while at boarding school. His teacher mixed aqueous solutions of magnesium sulfate and sodium bicarbonate and boomed at the class, “Behold the chaos! And the spirit of God hovered over the waters, and water and land separated.”^46^ [Quotations from the Dutch autobiography were translated to English by a machine [deepl.com] and evaluated by a native Dutch author (WLN).] Harting’s teacher was fixed on biblical metaphors because he knew no chemistry. Young Pieter, however, puzzled over the separation of the precipitate, about which he recalled “I decided not to rest until I understood the matter.”^46^ Curiously, Harting’s autobiography gives nary a mention of this indefatigable commitment. There is no recounting of his crystallization research (except in one letter about silver citrate in an exhaustive appendix of original documents). In large measure, the autobiography emphasizes his public disputes while serving on innumerable committees and commissions. It expresses admirable sentiments in places, and regrettable ones elsewhere. In any case, what is memorable to contemporary scientists and engineers, biomimetic crystallization, was forgettable to him at the end of his life.

### Calcospherites

Whether Harting was aware of Rainey or not, in introducing his major work on biomimetic crystallization, Harting said, “Everyone knows that [calcium carbonate], truly protean, occurs in extremely varied forms. . .[s]ometimes in the amorphous state, sometimes in the crystalline state, either of calcite or aragonite.”^43^ By contrasting “amorphous” with two crystalline phases, it seems clear that Harting did indeed recognize that there was an isotropic phase of calcium carbonate. Harting used the polarizing light microscope extensively in his analysis of so-called *calcospherites*, but it was to confirm the presence of radial crystalline bodies by the appearance of an extinction cross. He did not use light in his confirmation of the amorphous state. Nevertheless, ACC has been synthesized and characterized in recent times from bile and albumen solutions,^71,107^ conditions very similar to Harting’s in hindsight. There is every reason to believe that Harting, in 1872, also made lots of ACC. Indeed, Harting’s calcospherites were long recognized for the insights they might provide on mollusk shell formation. In 1902, Wilhelm Biedermann (1852–1929) evoked the appreciation of Harting that we heard above from Ozin:

[A]ll theorizing about the various possibilities of explaining the wonderful shell structures of mollusks appears to be a rather fruitless task as long as no further experience has been gained. However, the right way to do this was, it seems to me, shown a long time ago by Harting.^43^ As early as 1872, this researcher drew attention to the peculiar forms that calcium carbonate...takes when it is formed by the slow chemical interaction of two salts in a medium containing colloidal organic substances such as egg white, gelatin, mucus, etc. If you put a few pieces of CaCl_2_, on the one hand and a few crystals of NaCO_3_, on the other hand in two opposite places on the wall of a flat dish filled with egg white, after a few days a thin film forms from the edges, which gradually increases in thickness. After about 2 weeks, the skin appears to be made up of nothing but small spheres...^108^

Biedermann in Germany did not seem to know of Rainey.

### Politics and Social Transformation

In 1848, Harting joined the Society for the Abolition of Alcohol. In his autobiography 25 years later, he admitted that “his expectations have been realized only to a small extent.”^46^ This work morphed into a democratic association aimed to raise funds to help the poor but corruption by “political schemers” foreclosed on good intentions. Harting said that these experiences, “cured me for good of the political fever, which can be especially disastrous for a scientist.”^46^ This is not precisely true. While Harting may have avoided elective office, he maintained an interest in political causes throughout his life.

In 1870, Harting’s mood declined during the catastrophic Franco-Prussian war: “I deplored the war, I deplored those who caused it, ... I deplored my own insignificance, which made me completely powerless in the face of so much suffering.”^46^ Fighting his impotence, Harting wrote to Victor Hugo (1802–1885) because of the latter’s well-known abhorrence of war. Harting encouraged Hugo to persuade his compatriots to settle for peace but shortly thereafter Hugo published an open message urging the French to press on. Harting felt as if he had miscalculated.

In fact, Harting was hoping that the French would lie down in the face of what he perceived as Prussian superiority.^109^ Harting, a Darwinist, believed that ‘might makes right.’ Harting earned his detractors, especially others who considered themselves Darwinists such as the biologist Feringa who wrote in 1873: “...Harting’s Darwinism is a Darwinism *sui generis*. With his brand of Darwinism, which strongly resembles an abbatoir theory, one can defend not only an unjust war... but pretty well all forms of knavery.’’^109^

Hegeman notes that Feringa “defied Harting to prove that mankind could not progress under democracy,”^109^ a challenge that would be timely if reissued.

Harting’s prominence in Dutch society rose when in 1880 he published^110^ an open letter to the dissolved Committee to Restore Control of the Transvaal to Settlers of Dutch Descent, following the annexation of this region in 1877 by the British Empire. He chaired a national campaign for Transvaal independence^111^ protesting the annexation.^112^ A town in the Transvaal famous for hot springs was known for a while as Hartingsburg in honor of the biologist. Afterward, it was renamed Warbad (warm baths) but today is known as Bela–Bela (boiling pot in the Tswana language) about 165 km north of Johannesburg.

Some less affiliative sentiments by today’s standards were expressed by Harting in his autobiography and they can be found by the interested reader.

### Popular Writing

In 1852, Harting, with others, took on the job of editing a new journal, *Album der Natuur*,^113^ a popular science periodical with the translated subtitle “A work for the dissemination of natural knowledge among civilized readers of all classes.” Harting remembered having “devoted a fairly large portion of my time to our Album and always did so with particular pleasure.”^46^ He contributed articles on the following, in addition to many others not listed: Bioluminescence (1852), Hail (1853), Cork and corking (1855), A city of the dead in North America (1856), The origin of pearls (1857), (This article is necessarily a popularization of biomineralization. Pearls are polycrystalline aragonite.), The chameleon (1858), Photography (1859), Smithson’s foundation in Washington (1864), The mammoth (1867), Do animals think? (1869), A new method of preserving meat (1871), Do ducks have a keen sense of smell? (1872), Hallucinations and related phenomena (1873), A fight between a hyena and a man. Postscript (1873), Vivisection (1874), International Arctic research (1882), Transvaal gold (1884), Knowledge of nature as a means of education; a parting word (1885). Pieter Harting had a lot to say about a lot of things. For a full accounting of the dozens of additional essays, see Hubbrecht.^114^

Harting was not only interested in popularizing science, he fictionalized it too. In 1865, he published a novel forecasting the future, *Anno Domini 2065*.^115^ When translated to English six years later, the title was changed to *Anno Domini 2071*(116) to maintain a forward look of 200 years. Here, Harting tells of being escorted through Londinia by Roger Bacon (1220–1292), and his companion, Miss Phantasia. Harting admired Bacon because he too forecast the future: “Is it possible,” asked Bacon, “to construct spying-glasses by which the most distant objects can be drawn near to us, so that we shall be able to read the most minute writing[?]”^116^ The AFM is something like that, an innovation no one foresaw when Ward studied for his Ph.D. (1981).

Harting begins with a question suited to our time: “When comparing the present condition of society with that of past centuries the question naturally arises, what will the future be? Will the same progress which, in our own times especially, has been of such vast dimensions, and manifested itself in so many directions, *continue to be progressive*?”^116^ [ital. in original] At the time of writing, late 2024, who can say?

Harting forecast the age of aluminum, arriving sometime in the second half of the 20th century, whereas methods for extracting large quantities of aluminum metal from ore arrived at the close of the 19th century. The newfangled metal nevertheless inspired Harting to imagine great glass atria supported by lightweight alumina, warming in winter while freeing inhabitants from smoke, ash, and dust of indoor fires.

Miss Phantasia gives a spirited defense of art, not really obsolete in the age of photography as Harting, the time-traveler, feigned.

The National Library was forecast to be so large, one would have to choose subjects to investigate very narrowly, so as not to be exhausted by hiking. Interested in entomology? Choose just one order of insects in advance, advises Bacon. Who could have imagined the Internet before the Internet?

*Anno Domini* grabs attention as would a scorecard. Harting had hits and misses. But to the modern ear, accustomed to a great range of science fiction, Harting’s novel is dreadful from the first word to the last. The reputation of Jules Verne (1828–1905), a near contemporary, is secure.

### Giant Squid

**Figure 6:**
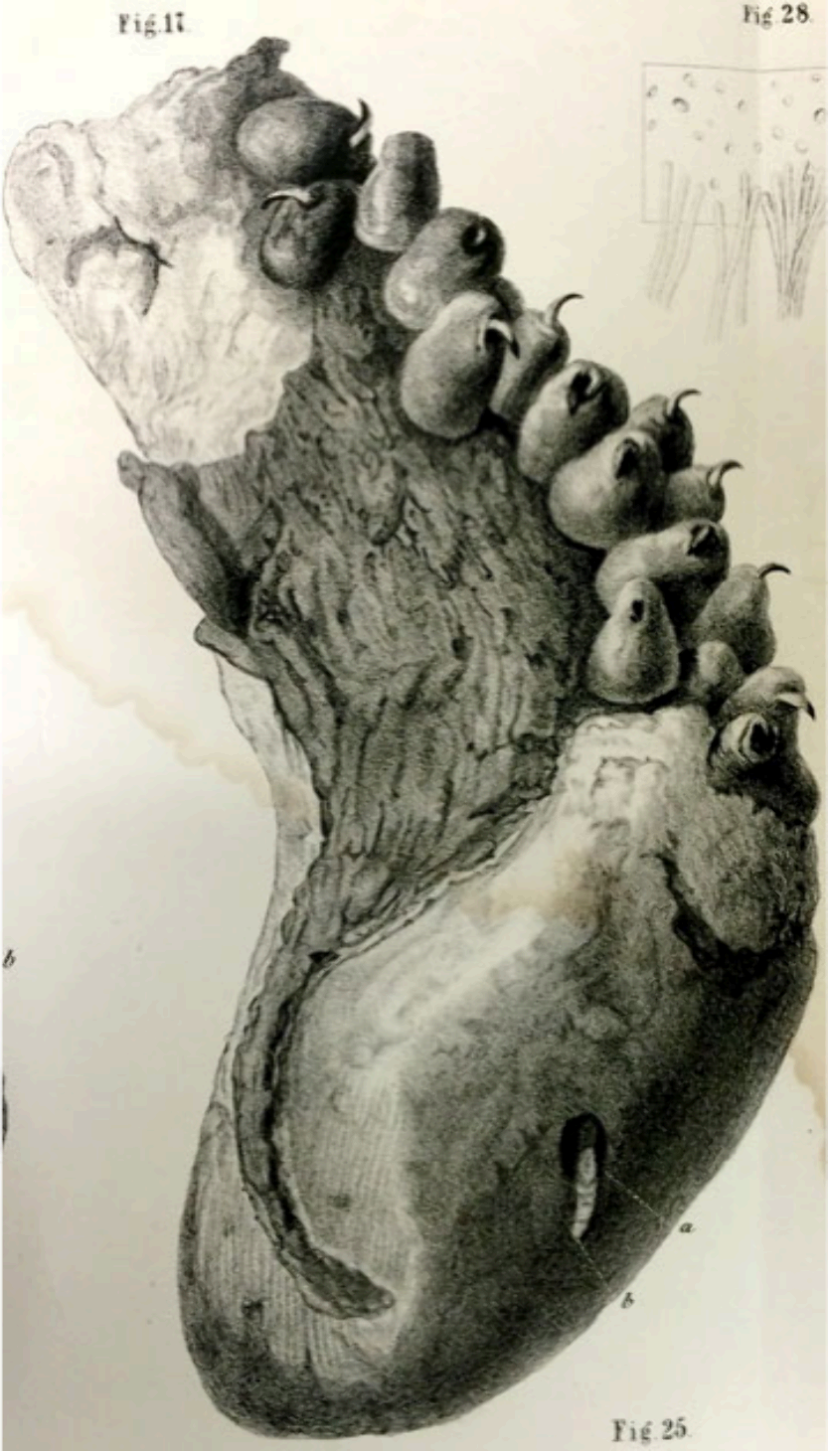
Giant squid fragment reproduced from ref (117). This work is in the public domain in the United States because it was published (or registered with the U.S. Copyright Office) before January 1, 1929.

If ever given the opportunity to discuss giant squid, take it. As a professor of zoology, Harting had examined the remains of two giant cephalopods (Figure 6).^117^ In honor of these observations, the Yale zoologist, Addison Verrill (1839–1926) named a species of giant squid *Loligo hartingii* after Harting.^118^ Today zoologists class Harting’s squid in the genus *Architeuthis*.^119^

## Summary

It cannot be doubted that George Rainey prepared ACC often from 1849 to 1861 and that Pieter Harting did likewise in 1872 more than 10 years later. Rainey’s procedure, repeated here and supported by modern methods of analysis, confirm the earlier conjectures. In many respects, the precipitation in stages of ACC *in vitro* in the presence of resins as described by Rainey resembles the comparable syntheses described by contemporaries with synthetic and naturally occurring polymers. Unlike “shy,” “retiring,” and “unobtrusive”^74^ Rainey, Harting persistently pressed against the grand themes of the times in which he lived. As a biomimetic crystallographer, among his many other interests and activities, he surely made metastable, ACC, and deserves recognition for these efforts, even if George Rainey had already done likewise.

Ward recognized that Science is a magnificent story that is international, intergenerational, and interdisciplinary. And, that it is a story that requires maintenance. He exercised this vigilance in the service of the community as a researcher, administrator, and ACS editor for decades. Here, we have done some recalibration with respect to Rainey and Harting. Such efforts enrich most any story, in our view, and ensure the fidelity of future measurements.
